# Highlighting peritumoral areas in human skin cancer biopsies by infrared micro-spectroscopy

**DOI:** 10.1186/1746-1596-8-S1-S33

**Published:** 2013-09-30

**Authors:** David Sebiskveradze, Cyril Gobinet, Valeriu Vrabie, Pierre Jeannesson, Olivier Piot, Michel Manfait

**Affiliations:** 1 MéDIAN, CNRS FRE 3481 MEDyC, SFR Cap-Santé, Université de Reims Champagne-Ardenne, 51 rue Cognacq-Jay, 51096 Reims, France; 2 CReSTIC, Université de Reims Champagne-Ardenne, Chaussée du Port, 51000 Châlons-en-Champagne, France

## Background

Fourier transform mid-infrared (FT–IR) microspectroscopy is a label-free optical method based on the interaction between an incident light beam and matter. This vibrational spectroscopy permits to probe the biochemical composition of the analyzed sample and thus gives information about the structure of this sample. Associated with an imaging system, FT–IR microspectroscopy of human tissues can be used as a very sensitive, non-destructive and non-subjective tool for the detection and localization of tumoral areas independently of visual morphology. Thus, FT–IR microimaging has demonstrated potential to provide clinically relevant diagnostic information in oncology [1-3].

The biochemical changes related to carcinogenesis between cancerous and surrounding tissue areas are subtle. As a consequence, IR hyperspectral images need to be processed by powerful digital signal processing and pattern recognition methods in order to highlight these changes [4,5]. To this end, an innovative fuzzy C-means (FCM) clustering-based algorithm was proposed in [6]. The real advantage of FCM is that it introduces the notion of nuance into the clustering of IR image pixels. Consequently, FCM allows considering the progressive transition between non-cancerous tissues and cancer lesions and reveals every nuance of intratumoral heterogeneity [6]. Moreover, the FCM-based algorithm proposed in [6] is fully automatic, i.e. the optimal clustering parameters such as the number of clusters are automatically determined. The main drawbacks with this algorithm are that it is very time consuming and that the transition areas can be difficultly seen. In this work, we thus propose solutions to these problems.

## Material and methods

IR spectral images were acquired on 8 biopsies of formalin-fixed paraffin-embedded human skin carcinomas, squamous cell carcinomas (SCC, n=3) and basal cell carcinomas (BCC, n=5). The samples were selected by the pathologists from the tumor bank of the Pathology Department of the University Hospital of Reims (France).

From samples, 10-micron thick slices were cut and mounted on a calcium fluoride (CaF_2_) (Crystran, Dorset, UK) window for FT–IR imaging without any particular preparation, especially no chemical dewaxing. First adjacent slices (5-µm thick) to those used for FT–IR analysis were stained with hematoxylin and eosin (H&E) for conventional histology. From these slices, the cancer outlines defined by the pathologists were drawn on the photomicrographs.

FT–IR hyperspectral images were recorded with a Spectrum Spotlight 300 FT–IR imaging system coupled to a Spectrum one FT–IR spectrometer (Perkin Elmer Life Sciences, France), with a spatial resolution of 6.25 µm and a spectral resolution of 4 cm^-1^. Each spectral image, covering a substantial part of the biopsy, consisted of about 30 000 spectra.

The samples being analyzed without previous chemical dewaxing, the recorded FT–IR hyperspectral image were digitally corrected from paraffin spectral contribution thanks to an automated pre-processing method based on extended multiplicative signal correction (EMSC) [7]. Only the spectral variability of the molecular composition of the tissue is thus retained in the data sets.

In order to highlight the different biological structures of the samples from the weak inter-spectra differences, the EMSC-based pre-processed IR spectra were analysed by an upgraded version of the FCM clustering-based algorithm proposed in [6]. The innovations mainly consist in the breaking of the FCM algorithm as soon as the estimated clusters present some uninteresting characteristics and in the limitation of the number of computed FCM. These computational aspects will be described later in details in another article. Furthermore, in order to highlight the transition areas between different tissue structures, an entropy based interconnectivity measure between clusters has been defined and applied on the FCM results. For cluster assignment, each color-coded map was then provided to the pathologists for a comparison with the corresponding H&E-stained sections.

## Results and discussion

After the application of the upgraded version of the FCM-based algorithm, the reconstructed color-coded clustering images allow recovering different histological structures automatically, particularly to precisely localize tumoral areas and their normal counterparts. Due to the limited length of the article, here we present only one representative case of SCC.

**Figure 1:**
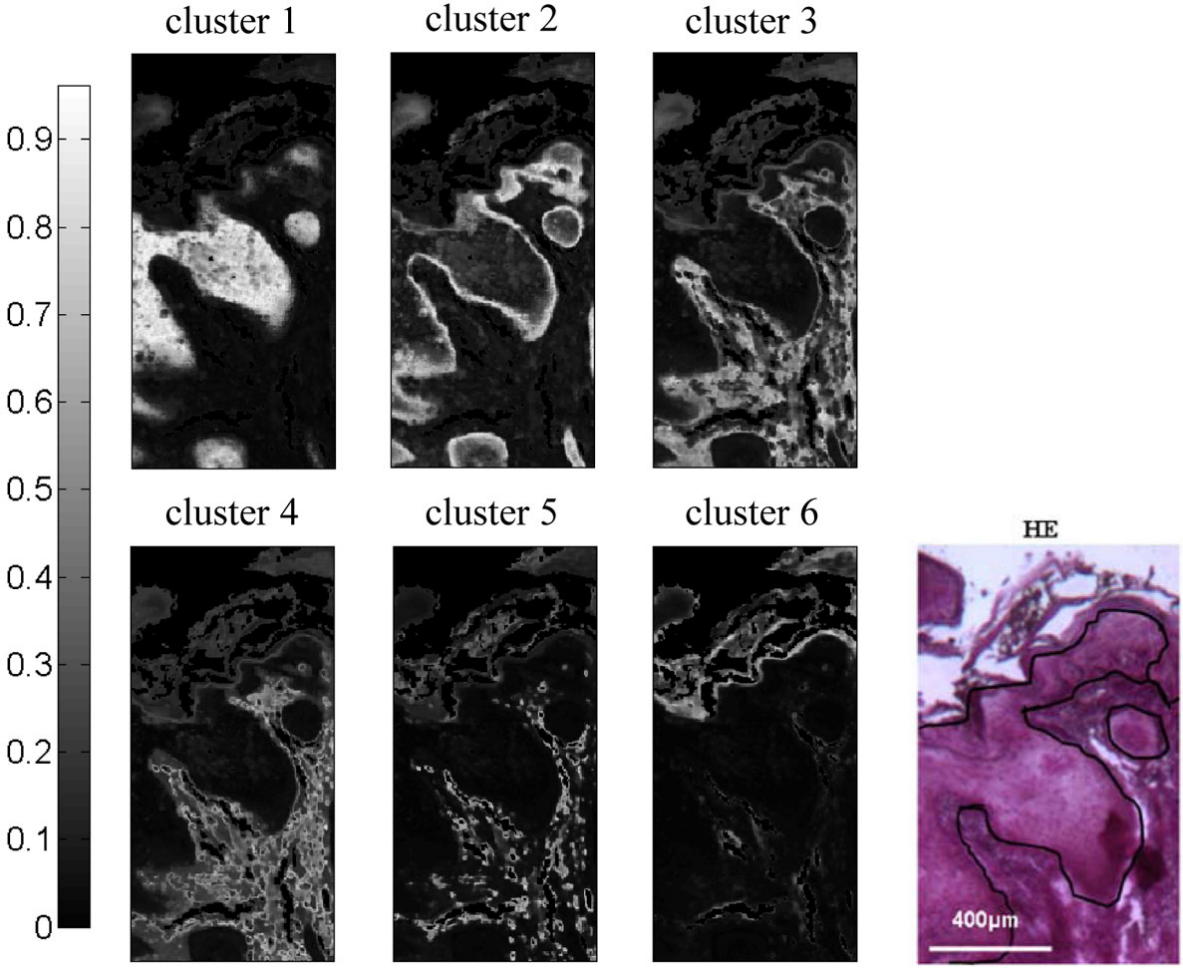
**Upgraded Fuzzy C-means (FCM) images on the Fourier transform mid-infrared (FT–IR) data set of the human skin squamous cell carcinoma (SCC) sample and the corresponding hematoxylin and eosin (H&E)-stained section.** Assignment of the clusters: 1 (tumor); 2 (invasive front); 3, 4 and 5 (dermis); 6 (epidermis). The color bar represents the membership value for each pixel. In the corresponding H&E-stained section, SCC is outlined.

The images generated by the FCM-based algorithm are shown in Figure 1. After comparison with the histological image, each generated cluster can be assigned to a precise tissue structure: tumoral area (cluster 1), invasive front (cluster 2), dermis (clusters 3, 4 and 5) and epidermis (cluster 6). These results are identical to those presented in [6], except that the computational time is divided by 8 thanks to the innovations included into the original algorithm.

**Figure 2:**
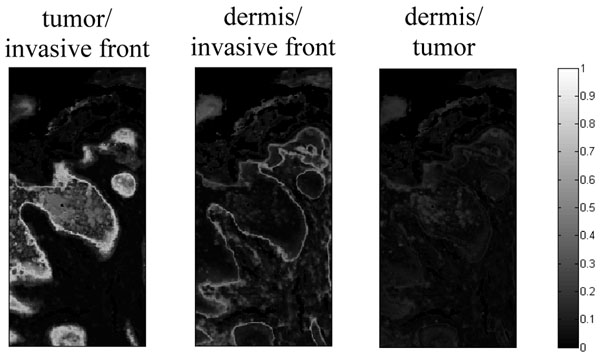
Images representing the entropy based interconnectivity measure computed on the upgraded Fuzzy C-means (FCM) images estimated on the SCC sample from Figure 1.

More than reproducing classical histology, our algorithm can give access to additional information about the assignment of the IR image pixels to the tissular structures. For each pixel, fuzzy clustering provides membership values, permitting to nuance their assignment. Such data are very valuable for the pixels located at the interface between tumoral tissue and its microenvironment. However, to ease the interpretation of transitional areas between tumor and marginal normal tissue, we developed an entropy based interconnectivity measure which is maximal when a pixel equally belongs to two clusters. Applied on the SCC IR image as shown on Figure 2, this interconnectivity measure shows that the invasive front (cluster 2) is tightly connected to the tumoral area (cluster 1) and that a surprising clear-cut difference between the invasive front (cluster 2) and the surrounding dermis (clusters 3, 4 and 5) exists.

These areas cannot be identified on hematoxylin-eosin staining or by conventional clustering of IR data.

## Conclusions

IR spectral microimaging associated with clustering techniques shows a great potential for the direct analysis of paraffin-embedded tissue sections of human skin cancers. These preliminary results show significant potential for probing tumor progression and for early determination of tumor aggressiveness in cutaneous cancers. Experiments are underway to define the molecular assignments of the spectral variations observed in these peritumoral areas. Furthermore, this approach could be applied to other human skin cancers such as melanoma.

## List of abbreviations

FT–IR: Fourier transform mid-infrared; FCM: fuzzy C-means; SCC: squamous cell carcinoma; BCC: basal cell carcinoma; H&E: hematoxylin and eosin; EMSC: extended multiplicative signal correction

## Competing interests

The authors declare that they have no competing interests.

## Authors' contributions

• DS realized the acquisitions of the data, developed the original FCM based algorithm, applied it on the IR images and drafted the extended abstract.

• CG developed the upgraded FCM based algorithm and the entropy based interconnectivity measure, and drafted the extended abstract.

• VV developed the original FCM based algorithm.

• PJ, OP and MM designed and managed the project
